# Microbial Diversity Analysis Using 16S rRNA Gene Amplicon Sequencing of Rhizosphere Soils from Double-Cropping Rice and Rice-Shrimp Farming Systems in Soc Trang, Vietnam

**DOI:** 10.1128/MRA.00595-21

**Published:** 2021-11-04

**Authors:** Thi-Xuan Do, Van-Phuc Huynh, Lan-Anh Le, Thuy-Vy Nguyen, Anh-Thi Nguyen-Pham, Minh-Dieu Bui-Thi, Anh-Thy Chau-Thi, Sy-Nam Tran, Van-Thanh Nguyen, Thuy-Duong Ho-Huynh

**Affiliations:** a Biotechnology Research and Development Institute, Can Tho University, Can Tho, Vietnam; b KTEST Science Company, Ho Chi Minh City, Vietnam; c Faculty of Agriculture, Can Tho University, Can Tho, Vietnam; d College of Environment and Natural Resources, Can Tho University, Can Tho, Vietnam; e Department of Genetics, Faculty of Biology and Biotechnology, University of Science, VNU-HCM, Ho Chi Minh City, Vietnam; University of Southern California

## Abstract

Different rice farming systems affect the soil microbial communities. Here, we report the results of 16S rRNA gene amplicon sequencing of soils collected from intensive rice cultivation and rice-shrimp farming systems in Soc Trang, Vietnam. The dominant phyla in these systems were *Firmicutes*, *Actinobacteriota*, *Chloroflexi*, *Myxococcota*, and *Acidobacteriota*.

## ANNOUNCEMENT

The Mekong Delta contributes 90% of the rice exports of the country. Due to global climate change, the intensive rice cultivation system along the coast of the region, developed 30 years ago, has been significantly changed into a rotational cropping system of rice-shrimp farming. The latter system has also been considered a sustainable system for agriculture in the coastal areas. Different rice farming systems affect the soil microbial communities (1–3), which play important roles in maintaining soil fertility and thus crop health and growth. However, the diversity and composition of the soil bacterial community in rice-shrimp farming systems remain unclear.

To address this issue, in this study, two rhizosphere soils were sampled from double-cropping rice (LL-DV) and rice-shrimp farming (LT-DV) systems at the beginning of the rice crop in September 2018. For each system, 10 soil core samples were randomly taken at depths of 0 to 15 cm and pooled into one sample, according to the method in our previous study (1). The chemical characteristics of the two soil samples representing the two investigated systems are described in Table 1. The two samples were kept at −80°C until metagenomic DNA extraction. DNA was extracted from 0.3 g of soil sample using the DNeasy PowerSoil kit (Qiagen, USA). Next, 16S rRNA gene amplicon sequencing libraries were prepared using the Swift amplicon 16S + internal transcribed spacer (ITS) panel (Swift Biosciences, USA) following the manufacturer’s guidance (8). Sequencing was performed at KTest Science Company, HCMC, Vietnam, using the Illumina MiSeq platform (2 × 150-bp paired ends). Adapters, primers, and low-quality sequences (average score, <20; read length, <100 bp) were removed using Trimmomatic version 0.39 (9) and Cutadapt version 2.10 (10). The reads were clustered and dereplicated into amplicon sequence variants (ASV) using the q2-dada2 plugin and denoise-single method within the QIIME2 pipeline version qiime2-2020.8 (11). Taxonomy assignment of the ASVs was performed using QIIME2 against the SILVA database version 138 SSURef Nr99 (12), with the q2-feature-classifier plugin and the classify-consensus-blast method (13). Functional profiles were predicted using PICRUSt2 version 2.3.0-b (14) and the MetaCyc database (4). Default parameters were used for all software unless otherwise specified.

**TABLE 1:** Chemical properties of soil samples corresponding to the two investigated systems

Sample	Location	pH^*a*^	Electric conductivity (mS/cm)^*a*^	Organic matter (%C)^*b*^	Total P (%P_2_O_5_)^*b*^	Total N (%N)^*c*^
LT-DV	9^o^32′18.3″N, 105^o^59′28.1″E	6.7	1.52	2.69	0.09	0.128
LL-DV	9^o^31′55.1″N, 105^o^59′51.7″E	5.5	0.62	3.02	0.07	0.163

a See reference 5.

b See reference 6.

c See reference 7.

**FIG 1:**
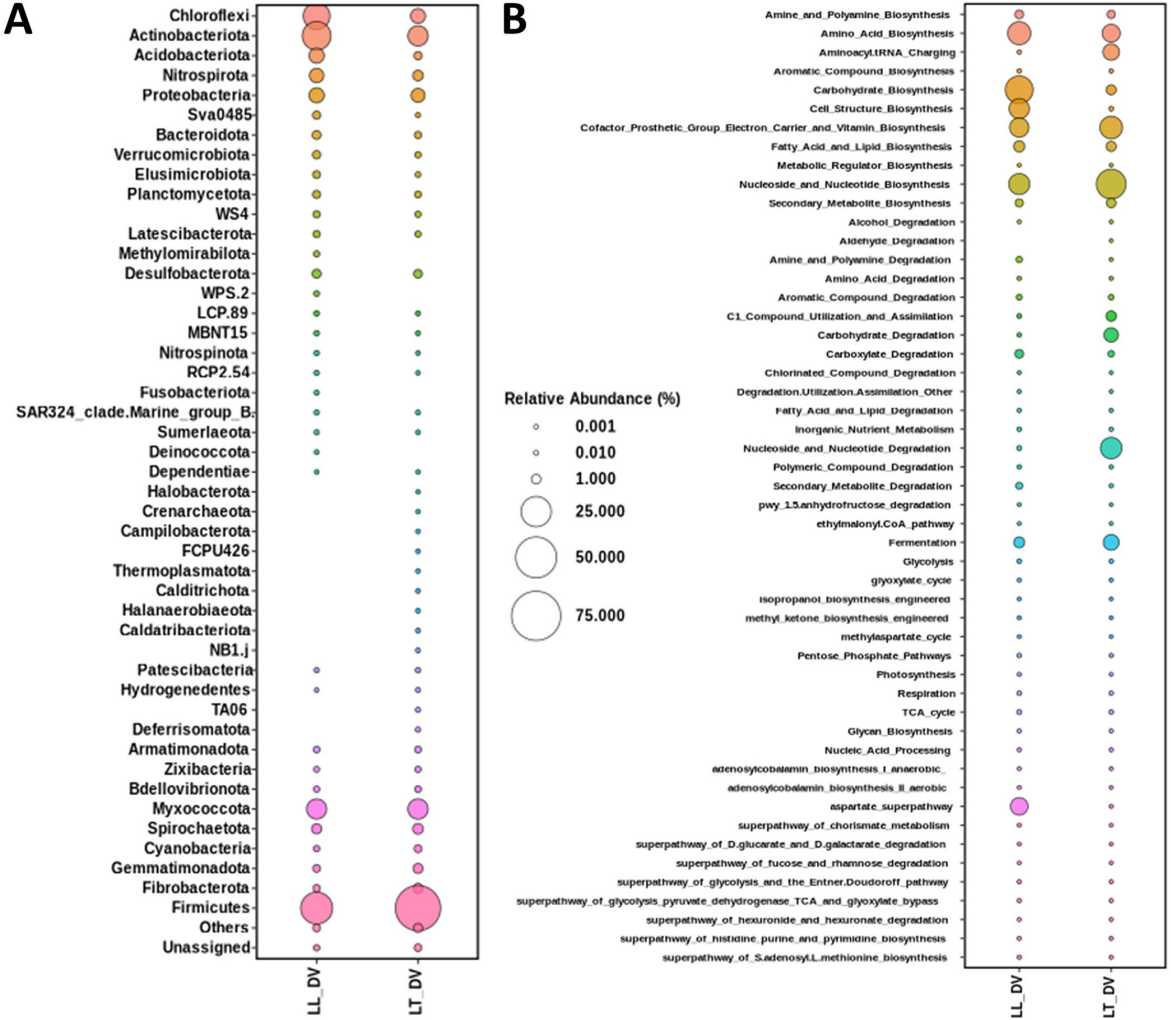
Taxonomic distribution at the phylum level (A) and functional analysis (B) based on 16S rRNA sequencing from two samples.

Taxonomic analysis at the phylum level and predictive functional profiles of the two bacterial communities are shown in Fig. 1A and B, respectively. The dominant phylum in both LL-DV and LT-DV was *Firmicutes* (28.6 and 62.9%), followed by *Actinobacteriota* (22.4 and 9.5%), *Chloroflexi* (18.8 and 4.1%), *Myxococcota* (9.4 and 9.5%), and *Acidobacteriota* (4.4 and 0.6%). This study is the first report on bacterial communities in rhizosphere soils from double-cropping rice and rice-shrimp farming systems using metagenomic next-generation sequencing. These results could be developed further to study the impacts of different soil microbial communities on rice cultivation.

### Data availability.

The 16S rRNA gene amplicon data sets have been deposited at DDBJ/ENA/GenBank under the accession number PRJNA725600 and can be accessed in the SRA under the accession numbers SRR14411138 (LL-DV) and SRR14411137 (LT-DV).
